# Identification, characterisation and outcomes of pre‐atrial fibrillation in heart failure with reduced ejection fraction

**DOI:** 10.1002/ehf2.15347

**Published:** 2025-06-18

**Authors:** Anna Helbitz, Ramesh Nadarajah, Lan Mu, Harriet Larvin, Hesham Ismail, Ali Wahab, Patrick Thompson, Peter Harrison, Mohammad Harris, Tobin Joseph, Sven Plein, Mark Petrie, Marco Metra, Jianhua Wu, Peter Swoboda, Chris P. Gale

**Affiliations:** ^1^ Faculty of Medicine and Health University of Leeds Leeds UK; ^2^ Leeds Institute of Data Analytics University of Leeds Leeds UK; ^3^ Leeds Institute for Cardiovascular and Metabolic Medicine University of Leeds Leeds UK; ^4^ Department of Cardiology Leeds Teaching Hospitals NHS Trust Leeds UK; ^5^ Wolfson Institute of Population Health, Queen Mary University of London London UK; ^6^ Department of Cardiology Mid Yorkshire NHS Trust Wakefield UK; ^7^ Institute of Cardiovascular and Medical Sciences University of Glasgow Glasgow UK; ^8^ Institute of Cardiology, ASST Spedali Civili di Brescia and Department of Medical and Surgical Specialties, Radiological Sciences, and Public Health University of Brescia Brescia Italy; ^9^ Department of Biomedical Imaging Science, Leeds Institute of Cardiovascular and Metabolic Medicine University of Leeds Leeds UK

**Keywords:** Atrial fibrillation, Cardiovascular magnetic resonance, Heart failure, Mortality, Prediction

## Abstract

**Aims:**

Atrial fibrillation (AF) in heart failure with reduced ejection fraction (HFrEF) has prognostic implications. Using a machine learning algorithm (FIND‐AF), we aimed to explore clinical events and the cardiac magnetic resonance (CMR) characteristics of the pre‐AF phenotype in HFrEF.

**Methods and results:**

A cohort of individuals aged ≥18 years with HFrEF without AF from the MATCH 1 and MATCH 2 studies (2018–2024) stratified by FIND‐AF score. All received cardiac magnetic resonance using Cvi42 software for volumetric and T1/T2. The primary outcome was time to a composite of MACE inclusive of heart failure hospitalisation, myocardial infarction, stroke and all‐cause mortality. Secondary outcomes included the association between CMR findings and FIND‐AF score. Of 385 patients [mean age 61.7 (12.6) years, 39.0% women] with a median 2.5 years follow‐up, the primary outcome occurred in 58 (30.2%) patients in the high FIND‐AF risk group and 23 (11.9%) in the low FIND‐AF risk group (hazard ratio 3.25, 95% CI 2.00–5.28, *P* < 0.001). Higher FIND‐AF score was associated with higher indexed left ventricular mass (β = 4.7, 95% CI 0.5–8.9), indexed left atrial volume (β = 5.9, 95% CI 2.2–9.6), higher indexed left ventricular end‐diastolic volume (β = 9.55, 95% CI 1.37–17.74, *P* = 0.022), native T1 signal (β = 18.0, 95% CI 7.0–29.1) and extracellular volume (β = 1.6, 95% CI 0.6–2.5).

**Conclusions:**

A pre‐AF HFrEF subgroup with distinct CMR characteristics and poor prognosis may be identified, potentially guiding interventions to reduce clinical events.

## Introduction

In patients with heart failure with reduced ejection fraction (HFrEF), the development of atrial fibrillation (AF) is associated with deterioration in quality of life, increased hospitalisation and worse prognosis.^1^ Data from the Framingham Heart Study show that heart failure is the strongest independent predictor of AF in both men and women,^2^ and it is estimated that between 30% and 40% of patients with heart failure will develop AF.^3^ Randomised clinical trial data show that aggressive rhythm control in patients with HFrEF and AF may improve outcomes.^4^, ^5^ Therefore, it might be clinically advantageous to identify individuals with HFrEF who are at high risk for onset of AF, such that they may be considered for heightened surveillance and targeted intervention to reduce incident AF.

Pre‐atrial fibrillation (AF) (stage 2 AF) defines a stage on the AF continuum characterised by structural or electrical findings predisposing to AF.^6^ There is limited information about the identification, characteristics and outcomes of individuals with HFrEF and pre‐AF. The Future Innovations in Novel Detection of Atrial Fibrillation (FIND‐AF) algorithm is a validated, scalable, routine electronic health records tool and calculator that predicts the risk of incident AF in the general population.^7^, ^8^ In this study, we investigated cardiovascular magnetic resonance (CMR) structural and functional parameters, clinical events and FIND‐AF risk scores in patients with HFrEF who did not have AF.

## Methods

### Study population

We retrospectively collected data from prospectively recruited patients enrolled in the MATCH (MyocArdial Tissue CHaracteristics in patients with heart failure according to glycaemic status) 1 and 2 registries who were clinically diagnosed with heart failure at cardiology clinics and referred for CMR assessment between 2018 and 2024 (MATCH 1 IRAS ID 228222 and MATCH 2 IRAS ID 273547). Patients were classified to have heart failure with reduced ejection fraction (HFrEF) if found to have a baseline LVEF ≤40% on echocardiography at the time of diagnosis. Patients were excluded if they had a baseline LVEF >40%, a known history of coronary artery disease (>70% on invasive coronary angiography), myocardial infarction (MI), coronary revascularisation or anginal symptoms. Other exclusion criteria included hypertrophic cardiomyopathy, amyloidosis, congenital heart disease and advanced renal failure. We only included patients from the MATCH registry who did not have AF at baseline.

### Patient characteristics

Clinical assessment, blood tests and CMR were carried out during one visit. Clinical information, including patient demographics, comorbidities and medication history, was obtained through direct interviews and routine electronic health records.

### CMR acquisition

Patients were advised to avoid caffeine intake for 24 h before CMR assessment. CMR images were acquired in a supine position using a 3T system (Siemens Magnetom Prisma, Erlangen, Germany). The study protocol included cine imaging, stress‐perfusion imaging and motion‐corrected bright‐blood late gadolinium enhancement (LGE). For stress imaging, adenosine was infused for at least 3 min, at a rate of 140 g/kg/min and if there was insufficient haemodynamic response (increase in heart rate <10 b.p.m.) or lack of symptomatic response, infused at an increased rate of up to 210 g/kg/min. LGE images were acquired as a short‐axis stack, and in four‐chamber, three‐chamber and two‐chamber views. When it was unclear if any enhancement on bright‐blood LGE represented ischaemic changes, a dark‐blood LGE stack was acquired.

### Image analysis

Image analysis was carried out on the cvi42 software (Circle Cardiovascular Imaging, Calgary Canada). LV volumes were measured by manual segmentation of endocardial and epicardial contours at end‐systole and end‐diastole.

The presence of LGE was confirmed if enhancement was identified on two orthogonal planes or, where available, on both bright and dark‐blood LGE images. Ischaemic LGE was defined as an enhancement that involved the subendocardium in a typical coronary distribution. Other patterns of fibrosis were categorised as non‐ischaemic apart from right ventricular insertion point fibrosis which was deemed not to have fibrosis. Inducible ischaemia was defined as the presence of visual perfusion defect affecting >1 myocardial segment on stress perfusion images not matched on rest perfusion or LGE images. CMR‐derived pulmonary capillary wedge pressure was calculated using published equations.^9^

### Find‐AF

Each patient's FIND‐AF score was derived using the previously published algorithm.^8^, ^10^ The FIND‐AF score predicts the likelihood of developing AF within 6 months for individuals aged ≥30 years based on routinely collected variables including age, sex, ethnicity and comorbidities.

### Study outcomes

The primary outcome was time to a composite of MI, stroke, heart failure hospitalisation and all‐cause mortality. Secondary outcomes included individual components of the primary outcome, AF and the association of CMR variables and FIND‐AF score.

### Statistical analyses

For baseline characteristics continuous variables are presented as mean ± SD, and *P* values are derived from two‐sample *t*‐tests. Categorical variables as frequencies and percentages, with *P* values determined from the chi‐square test.

To evaluate whether higher FIND‐AF scores were associated with more structural and functional abnormalities we conducted multiple univariate linear regression analyses for each CMR variable. We dichotomised patients into higher and lower FIND‐AF risk using a median split and examined the association with CMR variables with linear regression. Assumptions of linear regression were checked with diagnostic plots and we log‐transformed the CMR variables to improve normality. Scatter plots visualised the relationship between the FIND‐AF score and each CMR variable.

Wilcoxon test was used to examine whether there are differences in FIND‐AF scores between survival status. Univariate logistic regression was conducted to evaluate whether a higher FIND‐AF score is associated with an increased risk of death.

Kaplan–Meier plots show survival of outcomes with 10 or more events recorded stratified by higher and lower FIND‐AF risk scores defined by the median split in this population. Censor was applied on the date an outcome occurred following CMR, the date of chart review or the death date which comes earlier. Crude hazard ratios were calculated and adjusted for age.

Statistical analyses were performed in R, version 4.0.1 (R Foundation) with statistical significance set at *P* < 0.05. The study findings are reported following the Reporting of Studies Conducted Using Observational Routinely Collected Health Data (RECORD) recommendations and CODE‐EHR framework.^11^, ^12^

## Results

### Patient population

**Figure 1:**
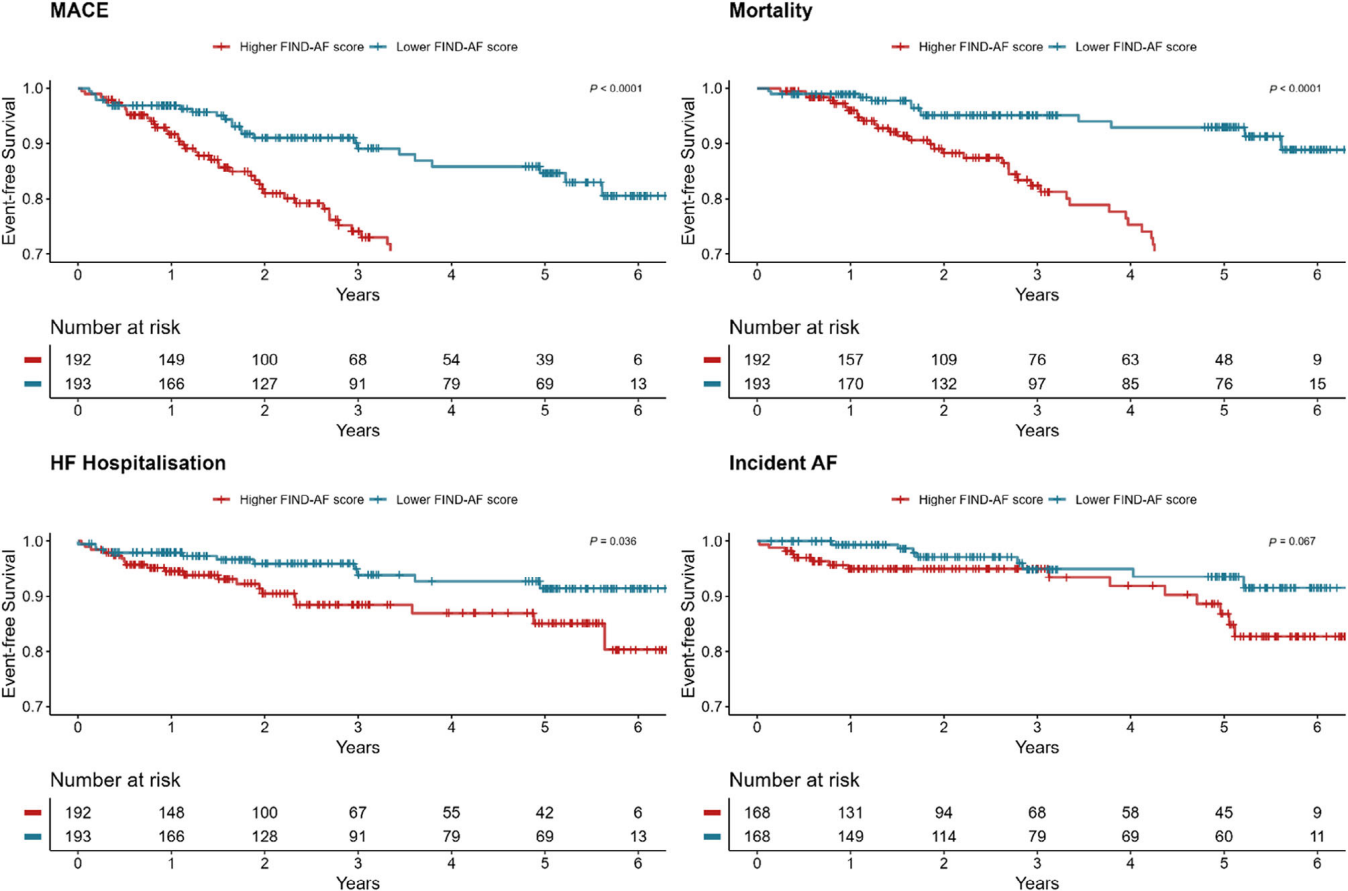
Patient event‐free survival by higher versus lower FIND‐AF risk for primary and secondary outcomes. AF, atrial fibrillation; HF, heart failure; MACE, major adverse cardiovascular event (composite of myocardial infarction, stroke, heart failure, hospitalisation, and all‐cause mortality).

Of 385 patients [mean age 61.7 (12.6) years, 39.0% women] with median 2.5 (IQR 1.3–5.1) years follow‐up (*Figure* *1*), we classified 192 to the high FIND‐AF risk classification and 193 to the low FIND‐AF risk classification. Patients with high FIND‐AF risk, compared with low FIND‐AF risk, were older (71.0 years vs. 52.5 years), more frequently had valvular heart disease (48.4% vs. 12.4%) and had a higher mean CHA_2_DS_2_‐VASc score (4 vs. 2). Additionally, patients with high FIND‐AF risk had significantly higher NT‐proBNP levels compared with those with low FIND‐AF risk [median 840 (IQR 1871) pg/mL vs. 199 (IQR 444) pg/mL, *P* < 0.001] (*Table* *1*).

**Table 1:** Baseline characteristics of the participants without AF by higher versus lower predicted risk of AF

	Total (*N* = 385)	Higher risk FIND‐AF score (*N* = 192)	Lower risk FIND‐AF score (*N* = 193)	*P* value
Age (years)	61.7 ± 12.6	71.1 ± 7.0	52.5 ± 9.8	<0.001
Women (%)	150 (39.0)	83 (43.2)	67 (34.7)	0.371
BMI (kg/m^2^)	28.7 ± 6.4	28.1 ± 6.3	29.4 ± 6.4	0.260
Hypertension (%)	174 (45.2)	101 (52.6)	73 (37.8)	0.112
Diabetes mellitus (%)	74 (19.2)	42 (21.9)	32 (16.6)	0.513
Vascular disease (%)	84 (21.8)	55 (28.6)	29 (15.0)	0.085
Chronic obstructive pulmonary disease (%)	38 (9.9)	27 (14.1)	11 (5.7)	0.173
Valvular heart disease (%)	117 (30.4)	93 (48.4)	24 (12.4)	<0.001
Stroke (%)	34 (8.8)	21 (10.9)	13 (6.7)	0.508
Chronic kidney disease (%)	18 (4.7)	13 (6.8)	5 (2.6)	0.392
Rheumatoid arthritis (%)	6 (1.6)	4 (2.1)	2 (1.0)	0.738
CHA_2_DS_2_‐VASc score	3.1 ± 1.4	3.9 ± 1.3	2.3 ± 1.0	<0.001
NTproBNP value	368 (1084.2)	840 (1871)	199 (444)	<0.001
Antiplatelet use	95 (26.2%)	58 (32.0%)	37 (20.3%)	0.016
Beta Blocker use	282 (77.7%)	145 (80.1%)	137 (75.3%)	0.327
Statin use	155 (42.7%)	98 (54.1%)	57 (31.3%)	<0.001
ACE inhibitor/angiotensin receptor blocker use	264 (72.7%)	136 (75.1%)	128 (70.3%)	0.362
Entresto use	55 (15.2%)	25 (13.8%)	30 (16.5%)	0.573
Mineralocorticoid receptor antagonist use	146 (40.2%)	66 (36.5%)	80 (44.0%)	0.178
Diuretic use	139 (38.5%)	77 (43.0%)	62 (34.1%)	0.101
Anticoagulant use	23 (6.4%)	14 (7.8%)	9 (4.9%)	0.374
SGLT2 inhibitor use	75 (20.6%)	41 (22.7%)	34 (18.6%)	0.406
Diabetes medication use	64 (17.7%)	34 (18.9%)	30 (16.5%)	0.644

### Outcomes

**Figure 2:**
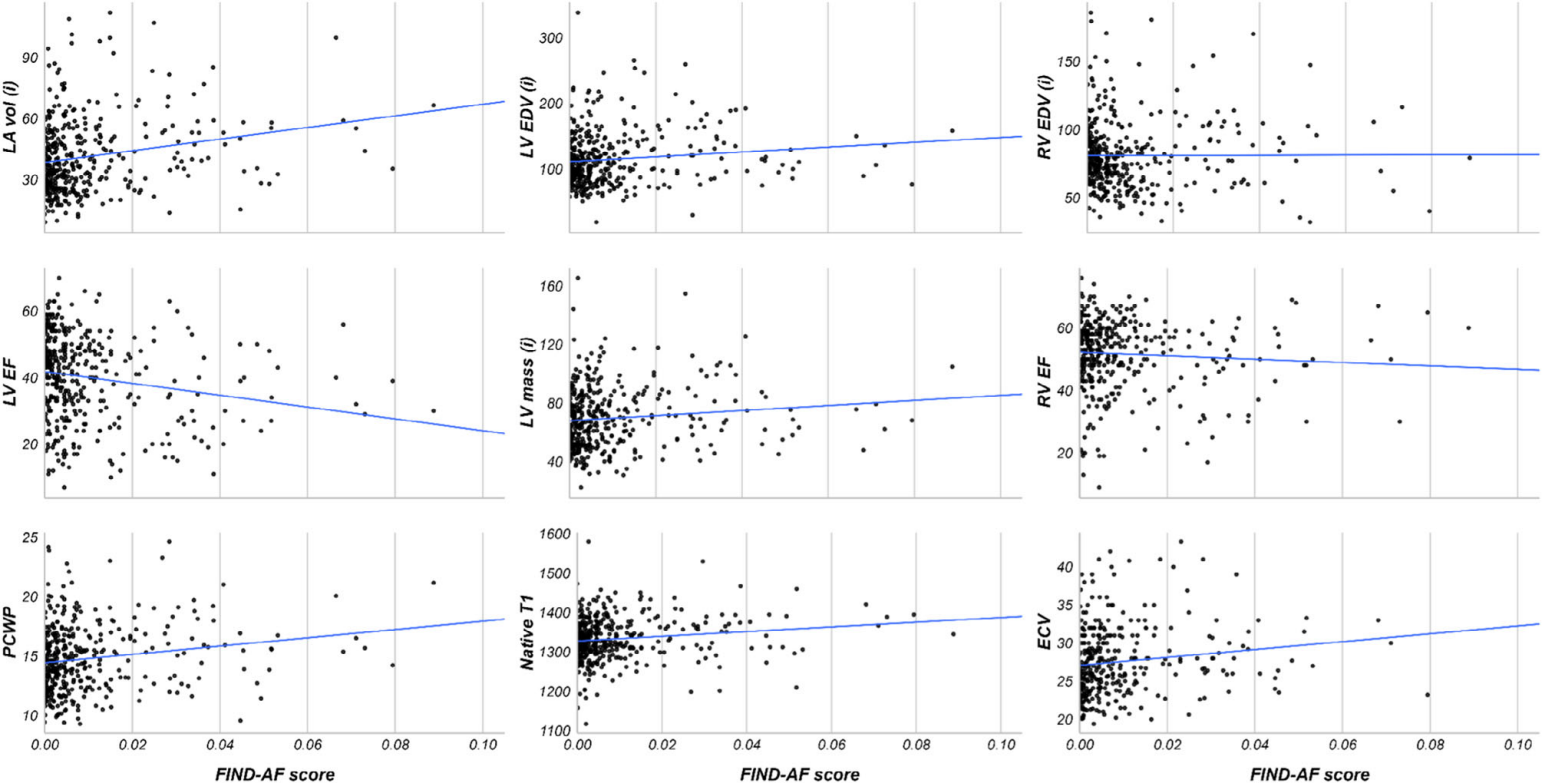
Distribution of FIND‐AF score by raw CMR parameters. ECV, extracellular volume; EDV, end‐diastolic volume; (i), indexed; LA, left atrial; LV, left ventricular; LVEF, left ventricular ejection fraction; RV, right ventricular; RVEF, right ventricular ejection fraction.

The primary outcome occurred in 58 (30.2%) patients in the high FIND‐AF risk group and 23 (11.9%) in the low FIND‐AF risk group (HR 2.89, 95% CI 1.35–6.20, *P* = 0.007) (*Figure* *2*). Death from any cause occurred in 42 patients (21.0%) in the high FIND‐AF risk group and 12 patients (6.0%) in the low FIND‐AF risk group (HR 4.38, 95% CI 2.30–8.33, *P* < 0.001), and heart failure hospitalisation occurred in 20 (10.4%) patients in the high FIND‐AF risk group and 11 (5.7%) patients in the low FIND‐AF risk group (HR 2.16, 95% CI 1.03–4.51, *P* = 0.041) (*Table* *S4*). When adjusted for differences in age between the groups, the risk of the primary outcome (HR 2.89, 95% CI 1.35–6.20, *P* = 0.006) and all‐cause mortality was still statistically significant (HR 6.74, 95% CI 2.42–18.74, *P* < 0.001) (*Table* *S5*).

Incident AF was diagnosed in 15 (7.8%) patients in the high FIND‐AF risk group and 8 (4.1%) patients in the low FIND‐AF risk group (HR 2.19, 95% CI 0.93–5.16, *P* = 0.074). Occurrences of MI and stroke were too infrequent individually for robust analysis (*Table* *S3*).

### CMR characteristics

Patients without AF with high FIND‐AF risk had lower mean LVEF (37.65 ± 12.66 vs. 42.53 ± 12.82, *P* = 0.0317) than those with a low FIND‐AF risk (*Table* *2*). Patients with high FIND‐AF risk also had double the rate of ischaemic fibrosis (27.6% vs. 14.0%, *P* = 0.0775) and inducible ischaemia [21 (10.9%) vs. 9 (4.7%), *P* = 0.263] than those with low FIND‐AF risk (*Table* *2*). Univariate linear regression demonstrated that a higher FIND‐AF risk was associated with lower LVEF (β = −4.88, 95% CI 7.44 to −2.31, *P* < 0.001), higher indexed LV end‐diastolic volume (EDV) (β = 9.55, 95% CI 1.37–17.74, *P* = 0.022), higher cMR‐derived pulmonary capillary wedge pressure (β = 0.69, 95% CI 0.13–1.25, *P* < 0.017), higher indexed LV mass (β = 4.68, 95% CI 0.50–8.86, *P* = 0.028), higher indexed LA volume (β = 5.87, 95% CI 2.16–9.59, *P* = 0.002), higher native T1 signal (β = 18.03, 95% CI 7.00–29.05, *P* = 0.001), and higher extracellular volume (β = 1.56, 95% CI 0.60–2.52, *P* = 0.001) (*Table* *3*). These patterns were consistent when the CMR parameters were log‐transformed (*Table* *S2*, *Figure* *S1*).

**Table 2:** CMR characteristics of the participants without AF by higher versus lower predicted risk of AF

	Total (*N* = 385)	Higher risk FIND‐AF score (*N* = 192)	Lower risk FIND‐AF score (*N* = 193)	*P* value
**Clinical cardiac magnetic resonance**				
Left atrial volume (mL)	79.8 ± 36.9	82.9 ± 39.2	76.8 ± 34.3	0.374
Left atrial volume indexed to BSA (mL)	41.3 ± 18.6	44.2 ± 20.3	38.4 ± 16.4	0.0941
Left ventricular ejection fraction (%)	40.1 ± 13.0	37.7 ± 12.7	42.5 ± 12.8	0.0317
Left ventricular mass (g)	135.5 ± 45.8	135.4 ± 45.4	135.6 ± 46.3	0.978
Left ventricular mass indexed to BSA (g/m^2^)	69.6 ± 20.8	72.0 ± 21.2	67.3 ± 20.3	0.211
Pulmonary capillary wedge pressure (mmHg)	14.8 ± 2.8	15.1 ± 2.8	14.4 ± 2.7	0.177
Right ventricular ejection fraction (%)	51.6 ± 11.1	51.3 ± 11.1	52.0 ± 11.1	0.705
Left ventricular end‐diastolic volume (mL)	222.8 ± 82.74	224.9 ± 86.26	220.7 ± 79.23	0.782
Left ventricular end‐diastolic volume indexed to BSA (mL)	114.9 ± 40.87	119.7 ± 43.65	110.1 ± 37.40	0.208
Right ventricular end‐diastolic volume indexed to BSA (mL)	81.01 ± 24.91	79.01 ± 25.98	83.03 ± 23.68	0.378
**Tissue characteristics**				
Ischaemic fibrosis (%)	80 (20.8%)	53 (27.6%)	27 (14.0%)	0.0775
Non‐ischaemic fibrosis (%)	114 (29.6%)	64 (33.3%)	50 (25.9%)	0.391
Inducible ischaemia (%)	30 (7.8%)	21 (10.9%)	9 (4.7%)	0.263
Native T1 (ms)	1333.0 ± 55.2	1343.0 ± 55.4	1324.0 ± 53.6	0.0662
Extracellular volume (%)	27.6 ± 4.6	28.4 ± 4.7	26.8 ± 4.3	0.0761

**Table 3:** Univariate linear regression showing the effect of higher FIND‐AF score on raw CMR variables in patients without AF

	β (95% CI)	*P* value
**Clinical cardiac magnetic resonance**		
Left atrial volume (mL)	6.07 (−1.36 to 13.5)	0.109
Left atrial volume indexed to BSA (mL)	5.87 (2.16–9.59)	0.002
Left ventricular end‐diastolic volume (mL)	4.18 (−12.48 to 20.85)	0.622
Left ventricular end‐diastolic volume indexed to BSA (mL)	9.55 (1.37–17.74)	0.022
Left ventricular ejection fraction (%)	−4.88 (−7.44 to −2.31)	<0.001
Left ventricular mass (g)	−0.22 (−9.45 to 9.01)	0.963
Left ventricular mass indexed to BSA (g/m^2^)	4.68 (0.50–8.86)	0.028
Pulmonary capillary wedge pressure (mmHg)	0.69 (0.13–1.25)	0.017
Right ventricular end‐diastolic volume indexed to BSA (mL)	−4.02 (−9.03 to 0.99)	0.116
Right ventricular ejection fraction (%)	−0.75 (−2.99 to 1.49)	0.512
**Tissue characteristics**		
Native T1 (ms)	18.03 (7.00–29.05)	0.001
Extracellular volume (%)	1.56 (0.60–2.52)	0.001

## Discussion

In this retrospective analysis of a prospective cohort, patients with HFrEF and pre‐AF, as defined by a high FIND‐AF risk, experienced a higher incidence of major adverse cardiovascular events, including all‐cause death and heart failure hospitalisation, compared to those with low FIND‐AF risk. CMR imaging revealed distinct structural and functional cardiac changes in the higher‐risk group, including reduced LV function, larger LV end‐diastolic volume, larger LA size, double the prevalence of silent MI, higher LV mass and a greater degree of diffuse myocardial fibrosis, as evidenced by higher T1 and ECV values.^13^ Our study demonstrates that patients with pre‐AF in HFrEF may benefit from closer monitoring and targeted interventions to prevent adverse outcomes.

Historical classifications of AF have been based on arrhythmia duration and emphasised AF once it had been diagnosed. The new classification of AF proposed in the recent American College of Cardiology/American Heart Association/American College of Clinical Pharmacy/Heart Rhythm Society guidelines incorporates a pre‐AF stage which recognises AF as a progressive disease and highlights the importance of prevention, risk factor management and screening in those at highest risk,^6^ and HFrEF is a well‐characterised high AF risk scenario. The prevention and early diagnosis of AF in patients with HFrEF is particularly important given that the co‐existence of these disease states is strongly associated with MACE. New‐onset AF more than doubles the mortality risk in those with HFrEF (HR = 2.72),^14^ and incident AF in patients with heart failure raises the risk of ischaemic stroke^15^ and is associated with heart failure hospitalisation and prolonged hospital stays,^16^ While previous studies have sought to predict the risk of AF in patients with HF,^17^, ^18^ none have assessed whether patients at higher predicted AF risk exhibit the pre‐AF phenotype and whether this has prognostic implications.

Inherent in the classification of pre‐AF is evidence of structural findings predisposing a patient to AF, and we found high FIND‐AF risk was associated with several of these changes in patients with HFrEF. Reduced left ventricular ejection function is related to elevated atrial pressures, neurohormonal activation, reduced parasympathetic tone and increased resistance to natriuretic peptides,^19^ which all contribute to AF development.^20^ We also found increased myocardial fibrosis, as evidenced by elevated T1 and ECV values, and LV mass, demonstrating the typical association between myocardial fibrosis, hypertrophy, and elevated filling pressures.^21^ Higher native T1 was associated with a 25% increased hazard of AF in a study of 42 308 patients in the UK Biobank.^22^ Higher ECV has been associated with an increased risk of AF recurrence after pulmonary vein isolation (HR 1.29, 95% CI 1.15–1.44). Left atrial dilatation not only causes structural alterations but also disrupts the normal propagation of electrical impulses, leading to modification in ionic currents.^23^ Furthermore, we found an increased prevalence of silent MI in patients with high FIND‐AF risk, pre‐AF, which can precipitate AF through the formation of re‐entry circuits due to shortened atrial effective refractory periods,^23^ non‐homogeneous cardiomyocyte conduction,^24^ and the development of atrial fibrosis following myocyte death and inflammation.^25^

Despite these structural changes that predispose to AF genesis, incident AF diagnosis in routine care was less common amongst patients with HFrEF and pre‐AF, even though the rate of MACE was very high. As incident AF diagnoses were taken from routine records, it is possible that some patients did not have AF episodes documented, and it is unlikely that patients underwent extended rhythm monitoring as part of routine care. A higher risk of MACE patients with high FIND‐AF risk was persistent even after adjustment for age differences, but it is uncertain whether these events were caused by the development of unrecognised AF or associated with the clustering of comorbidities incorporated within the FIND‐AF score that led to the manifestation of the pre‐AF substrate. Prospective assessment of patients with HFrEF and pre‐AF is required to ascertain the burden of subclinical AF in this population and explore whether this may be the causative step in adverse outcomes for these patients. Oral anticoagulation in patients with AF has been demonstrated to reduce the risk of stroke and all‐cause mortality.^26^ In patients with HFrEF, rhythm control of AF improves mortality and morbidity and significantly reduces the risk of stroke, hospitalisation for HF or acute coronary syndrome,^27^ and as such, it is recommended in the recent edition of the European Society of Cardiology guidelines.^28^ Patients with HFrEF and pre‐AF may have a higher burden of subclinical AF and are a subgroup who benefit from aggressive rhythm control or oral anticoagulation antecedent to the manifestation of clinical AF.

### Limitations

First, the patients in our study were real‐world patients with heart failure, and there may have been variety to the extent to which they were treated with optimal medical therapy. Second, CMR was performed in a single centre, but referrals were taken from multiple hospitals in the region, allowing for wide variations in treatment and patient factors, including social deprivation. Third, there were numeric differences in some of the CMR parameters and outcomes between the higher and lower predicted risk subgroups that did not reach statistical significance, and it may be that this reflects a Type II error. Fourth, in some patients who died during follow‐up, we were unable to access their primary care records to be able to identify whether they experienced AF before death, which led to fewer patients being included in that analysis and may have increased the risk of a Type II error. Fifth, death was identified from death certificates, and without access to original source data, we were unable to differentiate cardiovascular and non‐cardiovascular death. Sixth, we found that higher FIND‐AF risk was associated with structural and functional characteristics of pre‐AF in patients with HFrEF and that this has prognostic implications, but whether this extends to HFpEF is unknown. Seventh, inter‐atrial block was not systematically recorded and could not be included in our analysis. Given its known association with atrial fibrillation and adverse outcomes in heart failure,^29^ its absence may have limited our ability to fully capture AF risk. Finally, as the first study to apply an AF prediction algorithm in patients with HFrEF and evaluate both CMR characteristics and clinical outcomes in those at higher AF risk, future research is needed to validate these findings in external cohorts and assess their generalisability.

## Conclusions

In this cohort of patients presenting with HFrEF but without AF at baseline, pre‐AF, as defined by high FIND‐AF risk, was associated with lower left ventricular function, larger left ventricular and atrial volume, a diffuse myocardial fibrotic process, and major adverse cardiovascular events. The pre‐AF HFrEF cohort requires further study to identify if their higher rate of adverse outcomes is precipitated by subclinical AF and/or amenable to targeted intervention.

## Funding

None declared.

## Conflict of interest

All authors have completed the ICMJE uniform disclosure form at www.icjme.org/col_disclosure.pdf and declare: that CPG has received grants for research from Abbott and Bristol Myers Squibb; consulting fees from Astrazeneca, Bayer and Daiichi Sankyo; honoraria for speaking at meetings and educational events from Astrazeneca, Wondr Medical and Menarini; support for attending meetings from Bayer and Bristol Myers Squibb; and has acted as an advisory board member for Amgen, Astrazeneca, Bayer, Daiichi Sankyo and Menarini. MP has disclosed the following financial relationships: received grants from Boehringer Ingelheim, Roche, SQ Innovations, AstraZeneca, Novartis, Novo Nordisk, Medtronic, Boston Scientific, and Pharmacosmos; consulting fees from Boehringer Ingelheim, Novartis, AstraZeneca, Novo Nordisk, AbbVie, Bayer, Takeda, Corvia, Cardiorentis, Pharmacosmos, Siemens, and Vifor; and payment or honoraria for lectures, presentations, speaker bureaus, manuscript writing, or educational events from Boehringer Ingelheim, Novartis, AstraZeneca, Novo Nordisk, AbbVie, Bayer, Takeda, Corvia, Cardiorentis, Pharmacosmos, Siemens, and Vifor. MP has also served on Data and Safety Monitoring Boards or Advisory Boards for Teikoku and AstraZeneca and is a director of Global Clinical Trials Partners. All other authors declare no competing interests, or activities that could appear to have influenced the submitted work.

## Supporting information


**Table S1.** Components of the FIND AF Score
**Table S2.** Univariate linear regression showing the effect of higher FIND‐AF score on log‐transformed CMR variables
**Table S3.** Summary statistics for outcomes in Cohort according to FIND‐AF risk
**Table S4.** Crude hazard ratios of outcomes between higher and lower FIND‐AF risk
**Table S5.** Hazard ratios for outcomes between higher and lower FIND‐AF risk patients controlling for Age
**Figure S1.** Distribution of FIND‐AF score by log‐transformed CMR parameters

## Data Availability

Data are available on reasonable request.
