# Supplementary material for: Identification, characterisation and outcomes of pre‐atrial fibrillation in heart failure with reduced ejection fraction
Source: ESC Heart Fail. 2025 Jun 18;12(5):3688–96. doi: 10.1002/ehf2.15347 (PMC12450782; doi:10.1002/ehf2.15347)
Supplement: Supplementary file 1 — Table S1. Components of the FIND AF Score Table S2. Univariate linear regression showing the effect of higher FIND‐AF score on log‐transformed CMR variables Table S3. Summary statistics for outcomes in Cohort according to FIND‐AF risk Table S4. Crude hazard ratios of outcomes between higher and lower FIND‐AF risk Table S5. Hazard ratios for outcomes between higher and lower FIND‐AF risk patients controlling for Age Figure S1. Distribution of FIND‐AF score by log‐transformed CMR parameters [file EHF2-12-3688-s001.docx]

**Supplementary material**

Identification, characterisation and outcomes of pre-atrial fibrillation in heart failure with reduced ejection fraction

**Authors**

Anna Helbitz^1*^, Ramesh Nadarajah^2,3,4*^, Lan Mu^5^, Harriet Larvin^5^, Hesham Ismail^4^, Ali Wahab^2,3^, Patrick Thompson^3^, Peter Harrison^3^, Mohammad Harris^2,3^, Tobin Joseph^2,3^, Sven Plein^3^, Mark Petrie^7^, Marco Metra^8^, Jianhua Wu^5^, Peter Swoboda^9^, Chris P Gale^2,3,4^

*The first two authors are joint first authors.

**Table S1 Components of the FIND AF Score**

| **Variable** | **Description** |
| --- | --- |
| Age | Age of the patient |
| Sex | Code 1 if male, code 0 if female |
| Ethnicity | Code 1 if white, code 0 if other |
| Heart failure | Diagnosis of heart failure |
| Hypertension | Diagnosis of hypertension |
| Diabetes | Diagnosis of diabetes |
| Vascular disease | Diagnosis of vascular disease |
| Chronic obstructive pulmonary disease | Diagnosis of chronic obstructive pulmonary disease |
| Valvular heart disease | Diagnosis of valvular heart disease |
| Stroke | Diagnosis of previous stroke |
| Hyperthyroidism | Diagnosis of hyperthyroidism |

**Table S2** **Univariate linear regression showing the effect of higher FIND-AF score on log-transformed CMR variables**

|  | **β (95% CI)** | **p value** |
| --- | --- | --- |
| **Clinical cardiac magnetic resonance** |  |  |
| Left atrial volume (ml) | 0.06 (-0.03 - 0.16) | 0.18 |
| Left atrial volume indexed to BSA (ml/m^2^) | 0.13 (0.04 - 0.22) | 0.004 |
| Left ventricular ejection fraction (%) | -0.13 (-0.21 - -0.05) | 0.001 |
| Left ventricular mass indexed to BSA (g/m^2^) | 0.07 (<0.01 - 0.13) | 0.029 |
| Left ventricular mass (g) | <0.01 (-0.07 - 0.07) | 0.979 |
| Pulmonary capillary wedge pressure (mmHg) | 0.04 (<0.01 - 0.08) | 0.018 |
| Right ventricular ejection fraction (%) | -0.01 (-0.07 - 0.04) | 0.681 |
| Left ventricular end-diastolic volume (ml) | <0.01 (-0.07 - 0.08) | 0.889 |
| Left ventricular end-diastolic volume indexed to BSA (ml) | 0.07 (<0.01 - 0.13) | 0.061 |
| Right ventricular end-diastolic volume indexed to BSA (ml) | -0.06 (-0.12 - <0.01) | 0.035 |
| **Tissue characteristics** |  |  |
| Native T1 (ms) | 0.01 (<0.01 - 0.02) | 0.001 |
| Extracellular volume (%) | 0.06 (0.02 - 0.09) | 0.001 |

**Table S3** **Summary statistics for outcomes in Cohort according to FIND-AF risk**

|  | **Total (N=385)** | **Higher risk FIND-AF score (N=192)** | **Lower risk FIND-AF score (N=193)** |
| --- | --- | --- | --- |
| **MACE outcome** | 81 | 58 | 23 |
| **All-cause mortality** | 54 | 42 | 12 |
| **Heart failure hospitalisation** | 31 | 20 | 11 |
| **Incident AF** | 23 | 15 | 8 |
| **Myocardial infarction** | 3 | 3 | 0 |
| **Stroke** | 7 | 3 | 4 |

MACE defined as a composite of myocardial infarction, stroke, heart failure hospitalisation, and all-cause mortality

AF, atrial fibrillation

**Table S4: Crude hazard ratios of outcomes between higher and lower FIND-AF risk**

|  | **Hazard Ratio** | **95% CI** | **p value** |
| --- | --- | --- | --- |
| **MACE outcome** | 3.25 | (2.00, 5.28) | < 0.0001 |
| **Heart failure hospitalisation** | 2.16 | (1.03, 4.51) | 0.041 |
| **All-cause mortality** | 4.38 | (2.30, 8.33) | <0.0001 |
| **Incident AF** | 2.19 | (0.93, 5.16) | 0.074 |
| Note: Lower AF risk as reference level | | | |

MACE defined as a composite of myocardial infarction, stroke, heart failure hospitalisation, and all-cause mortality

AF, atrial fibrillation

**Table S5: Hazard ratios for outcomes between higher and lower FIND-AF risk patients controlling for Age**

|  | **Hazard Ratio** | **95% CI** | **p value** |
| --- | --- | --- | --- |
| **MACE outcome** | 2.89 | (1.35, 6.20) | 0.0064 |
| **Heart failure hospitalisation** | 0.78 | (0.25, 2.44) | 0.67 |
| **All-cause mortality** | 6.74 | (2.42, 18.74) | 0.00026 |
| **Incident AF** | 0.60 | (0.14, 2.46) | 0.47 |
| Note: Lower AF risk as reference level | | | |

MACE defined as a composite of myocardial infarction, stroke, heart failure hospitalisation, and all-cause mortality

AF, atrial fibrillation

**Figure S1 Distribution of FIND-AF score by log-transformed CMR parameters**


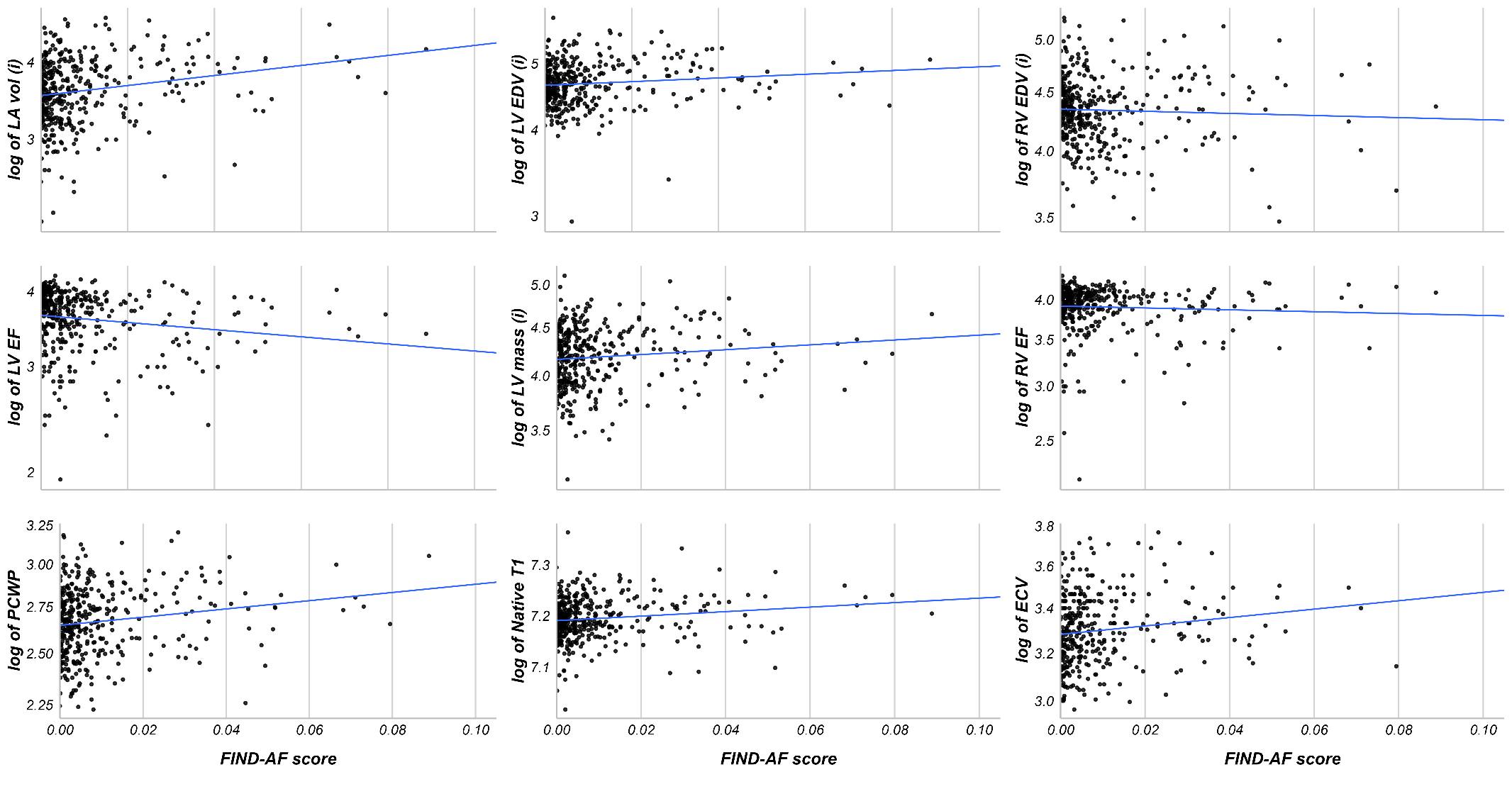


ECV, extracellular volume; LA, left atrial; LV, left ventricular; LVEF, left ventricular ejection fraction; RVEF, right ventricular ejection fraction; EVD, end-diastolic volume; (i), indexed.
